# Use of FCC-NMRD relaxometry for early detection and characterization of *ex-vivo* murine breast cancer

**DOI:** 10.1038/s41598-019-41154-9

**Published:** 2019-03-15

**Authors:** Enza Di Gregorio, Giuseppe Ferrauto, Stefania Lanzardo, Eliana Gianolio, Silvio Aime

**Affiliations:** 1 Department of Molecular Biotechnology and Health Sciences, Via Nizza 52, 10126 Torino, Italy; 2 IBB-CNR, Sede secondaria c/o Molecular Biotechnology Center, Via Nizza 52, 10126 Torino, Italy

## Abstract

Breast Cancer is the most diffuse cancer among women and the treatment outcome is largely determined by its early detection. MRI at fixed magnetic field is already widely used for cancer detection. Herein it is shown that the acquisition of proton T_1_ at different magnetic fields adds further advantages. In fact, Fast Field Cycling Nuclear Magnetic Resonance Dispersion (FFC-NMRD) profiles have been shown to act as a high –sensitivity tool for cancer detection and staging in *ex vivo* murine breast tissues collected from Balb/NeuT mice. From NMRD profiles it was possible to extract two new cancer biomarkers, namely: (i) the appearance of ^14^N-quadrupolar peaks (QPs) reporting on tumor onset and (ii) the slope of the NMRD profile reporting on the progression of the tumor. By this approach it was possible to detect the presence of tumor in transgenic *NeuT* mice at a very early stage (5–7 weeks), when the disease is not yet detectable by using conventional high field (7 T) MRI and only minimal abnormalities are present in histological assays. These results show that, NMRD profiles may represent a useful tool for early breast cancer detection and for getting more insight into an accurate tumor phenotyping, highlighting changes in composition of the mammary gland tissue (lipids/proteins/water) occurring during the development of the neoplasia.

## Introduction

Breast Cancer is a multifactorial disease, considered a major public-health issue worldwide and the second cause of cancer-associated death among women^1,2^. The majority of breast cancers develops over extended periods of time arising from early, pre-invasive lesions such as atypical ductal hyperplasia (ADH) and ductal carcinoma *in situ* (DCIS), progressing to invasive carcinoma (IC) and culminating in the metastatic disease^3^. Early diagnosis is considered crucial for the success of the applied breast cancer therapy^4^. Recent years have witnessed a great progress in early diagnosis of breast cancer and in the design of personalized treatments, specific for the various tumor phenotypes.

Several imaging techniques are available for diagnosis, namely mammography, magnetic resonance imaging (MRI), positron-emission tomography (PET), single-photon emission computed tomography (SPECT), Computed tomography (CT) and Ultrasounds (US)^5–9^.

Currently, mammography and/or US^10,11^ are the gold standard techniques used for population screening. More informative images can be obtained by using Magnetic Resonance Imaging (MRI), which provides detailed images of deep tissues, with a very high spatio-temporal resolution, without using ionizing radiation and with a limited invasiveness^5,12,13^.

Often, MR images are obtained without the administration of exogenous contrast agents, simply by exploiting the endogenous contrast generated by the differences in transverse and longitudinal water proton relaxation times (T_2_ and T_1_) between healthy and tumor tissues^14–16^.

We surmise that further, relevant information may be obtained by exploiting the T_1_ dependence of protons tissue from the applied magnetic field strength (B_0_). This task cannot be yet accomplished on the available MRI scanners, because they work at fixed B_0_. Nevertheless, the acquisition of the so called Fast Field Cycling–Nuclear Magnetic Dispersion (FFC-NMRD) profiles^17–19^ on *ex vivo* tissues can provide useful insights into the potential diagnostic value of Field-dependent T_1_-based approach^20^.

These profiles report about ^1^H water relaxation rate, R_1_ (where R_1_ = 1/T_1_), over an extended range of magnetic fields. Their analysis can provide useful information about tissue water dynamics and the presence of lipids, protein and paramagnetic species as well as the interaction of water with proteins and other macromolecules^14–16,19–21^.

In this study, BALB-neuT transgenic mice have been used as breast cancer models^22^. Transgenic mouse models of breast cancer provide evident advantages in preclinical studies compared to xenografted ones. In fact, the latter model appears more suitable to represent advanced stages of cancer, whereas transgenic mice recapitulate the stepwise progression and typical features of several human cancers, maintaining the characteristic interactions that naturally occur between tumors and their surrounding microenvironment^22^. BALB-neuT mice show a human-like breast cancer development, passing from mammary hyperplasia to atypical hyperplasia, and *in situ* carcinoma (CIS) and finally, to invasive breast cancer^3,22^. Breast tissue specimens from mice at different tumor stages of development have been analyzed by acquiring FFC-NMRD (Fast Field Cycling- Nuclear Magnetic Resonance Dispersion) profiles, without the administration of exogenous contrast agents (CAs). Correlations between the NMRD read-out and tumor stages/phenotypes have been assessed.

## Methods

### Animals

Female BALB/c mice (12-weeks) were purchased from Charles River Laboratories (Calco, Italy) and used as healthy control. BALB-neuT mice^22^ (5–30 weeks) were bred at the Molecular Biotechnology Center of the University of Torino (It).

Experiments involving animal testing were carried out at the Molecular Biotechnology Center at the University of Torino (Italy). Mice were kept in standard housing (12 h light/dark cycle) with rodent chow and water available *ad libitum*). Experiments were performed according to the Amsterdam Protocol on Animal Protection and in conformity with institutional guidelines that are in compliance with national laws (D.L.vo 116/92, D.L.vo 26/2014 and following additions) and international laws and policies (EEC Council Directive 86/609, OJL 358, Dec 1987; NIH Guide for the Care and Use of Laboratory Animals, U.S. National Research Council, 1996).

This study was carried out in the framework of a protocol approved by Italian Ministry of Health (n807/2017-PR 19/10/2017).

### MRI acquisition

For the MRI experiments, mice were anesthetized by intramuscular injection of a mixture of Tiletamine/Zolazepam (Zoletil 100, Virbac, Milan, Italy) 20 mg/kg and xylazine (Rompun; Bayer, Milan, Italy) 5 mg/kg.

MR images were acquired at 7.1 T on a Bruker Avance 300 spectrometer equipped with the Micro 2.5 microimaging probe at room temperature (R.T. = 21 °C).

High resolution T_2W_ images were acquired by using a RARE (Rapid Acquisition with Refocused Echoes) sequence with the following parameters: Repetition time (TR) = 4000 ms, Echo time (TE) = 36 ms, RARE factor = 8, flip angle (FA) = 180°, number of averages(NA) = 8, Field of view (FOV) = 40 mm × 40 mm, slice thickness = 0.5 mm, matrix size 384 × 384, spatial resolution = 0.104 mm/pixel × 0.104 mm/pixel).

### FFC-NMRD profiles acquisition and data analysis

Mice were sacrificed by cervical dislocation, in agreement with ethical European guidelines. All mammary glands were gently scraped from the skin and divided into two parts, respectively used for i) the acquisition of FFC-NMRD (Fast Field Cycling- Nuclear Magnetic Resonance Dispersion) profiles and ii) for the histological analysis.

Mammary glands at different tumor stage (5–7w, 15w, 21w, 30w) and mammary glands from healthy control mice (N > 8) were analyzed and data were reported as mean ± SD. Tissue were placed into 10-mm NMR tubes without the addition of any medium or buffer, in order to avoid the addition of water to the specimen.

Nuclear magnetic resonance dispersion profile (NMRD) were acquired at 25 °C over a continuum of magnetic field strength from 0.01 to 21.5 MHz proton Larmor frequencies (corresponding to B_0_ = 0.24 mT-0.5 T) on the Stelar SpinMaster FFC NMR relaxometer (Stelar S.n.c., Mede (PV), Italy). The signal is placed on resonance and tuned at the proton Larmor frequency of each analyzed magnetic field. The applied sequences were the Pre Polarized sequence (PP/S) between 0.01 MHz and 7 MHz and the Non-Polarized sequence (NP/S) between 7 and 21.5 MHz, respectively. Sixteen delay times (τ) between pulses were used. T_1_ was determined by applying the saturation recovery function and analyzed as a mono-exponential decay (Bloch equation). The reported longitudinal relaxation rate (R_1_) corresponds to 1/T_1_^23^. The total acquisition time is less than 50 minutes.

Analytical analysis of QPs (Quadrupolar peaks) was carried out according to mathematical models and procedures reported in literature by using Origin 9 software^24–26^.

The region containing the quadrupolar peaks was highlighted by subtracting the background of the R_1_ dispersion curve. The procedure relied on the fitting of the background using a bi-exponential model, which was found empirically to fit closely to the background data (*i.e*. experimental data in the regions not covering peaks’ region, B_0_ < 9 mT and B_0_ > 90 mT). The background contribution was then subtracted to the experimental NMRD profiles to make more visible the occurrence of QPs.

### Histological analysis

Specimens for histology were fixed in 10% Neutral Buffered Formalin (Bio-Optica, Milan, Italy) and then embedded in paraffin; 5 μm slides were cut and placed in glass slides for histology. Sections were deparaffinized in xylol, rehydrated in graded alcohol series (Sigma Aldrich, Milano, Italy), then washed in water and stained with Hematoxylin (BioOptica) and Eosin (BioOptica) (H&E) for histological examination. The slices were analyzed on an Olympus 41microscope.

### Statistical analysis

Data are represented as mean ± SD. A paired two-tail Student’s t-test was used for all the experiments.

### *In vitro* validation studies

The mixture of tissues (adipose and epithelial) present in mammary gland in the presence of tumor has been simulated *in vitro* by mixing cross-linked albumin (representative of epithelial tissue) and oil (representative of fat tissue).

Cross-linked albumin was obtained by using a well-established procedure^27,28^. Briefly, BSA (Bovine Serum Albumin, Sigma Aldrich) was dissolved in water (150 mg/mL) and the solution placed in a heated bath at 80 °C for 10 minutes. The heating led to the formation of a semisolid system containing immobilized cross-linked proteins. Then, the cross-linked BSA has been gently mixed with oil at different ratios (i.e. BSA/oil is 0, 0.1, 0.33, 0.50, 0.66, 1).

NMRD profiles have been acquired and analyzed as done for animal tissues.

## Results

*Balb-NeuT* mice have been investigated at different stage of their growth, *i.e*.at the age of (i) 5–7w, (ii) 15w, (iii) 21w and (iv) 30w, respectively. As control, healthy female Balb/c mice have been used. All mice underwent MRI scans to monitor the tumor growth.

**Figure 1:**
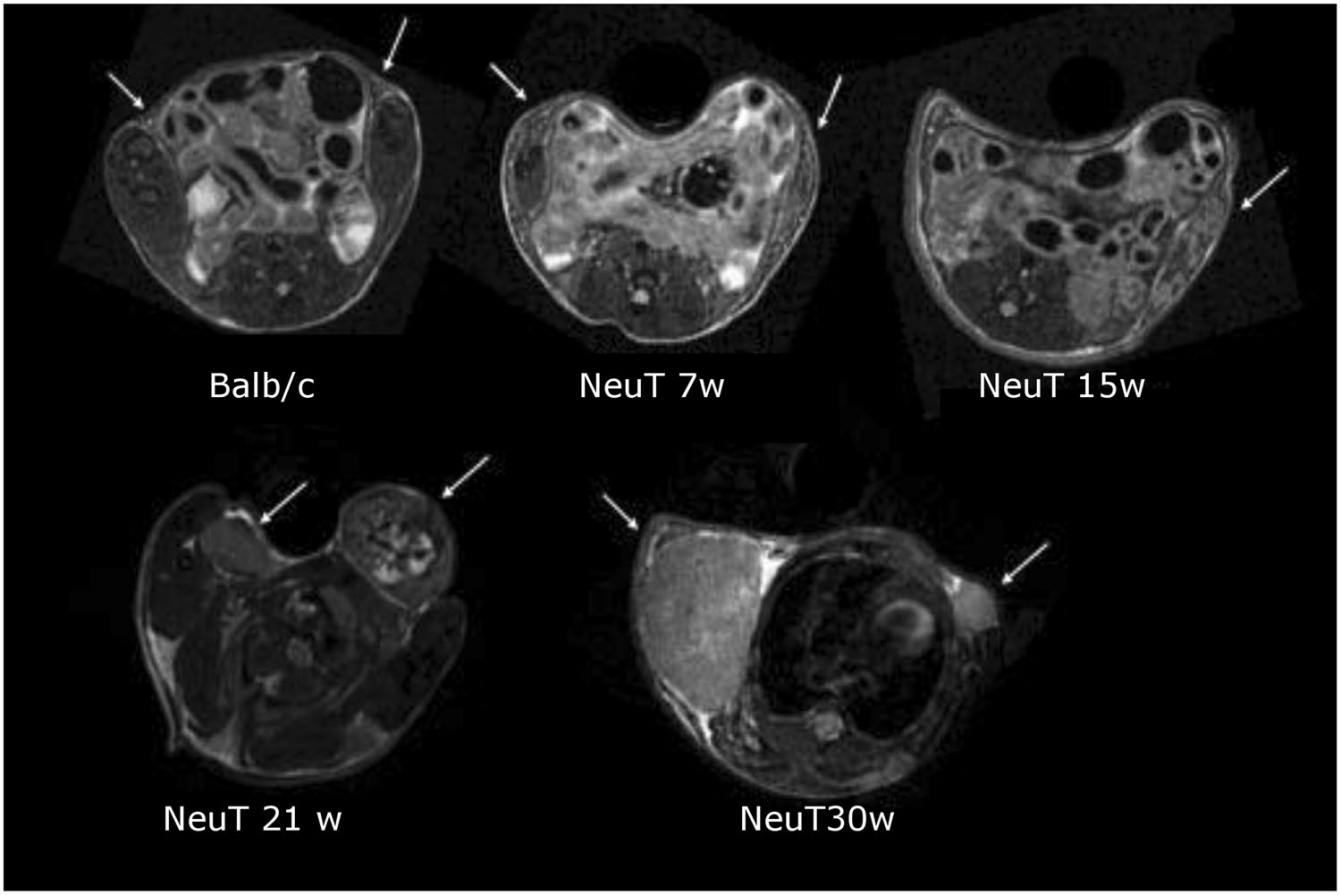
Axial T_2w_ MR Images of Balb/C-NeuT mice at different growth stages and health Balb/c control (white arrows indicates breast/tumor regions).

In Fig. 1 representative T_2w_ MR Images (axial slices) of mice at the four considered stages are reported and compared with control mice. In the first stage of development (5–7 week), tumor is not detectable by MRI. Breast displays the same morphology and MR contrast cannot differentiate diseased from healthy mice. At the age of 15 weeks, the presence of cancer cells embedded in the fatty matrix of the mammary gland tissue is barely detectable by MRI. Tumor becomes well visible in the MR images taken at 21–30 weeks, when the entire breast region is invaded by cancer cells. Therefore, the detection threshold by a typical MRI-T_2_ scan may be set at 15 weeks. At each time point, after the acquisition of MR images, mammary glands were excised and their NMRD profiles recorded.

**Figure 2:**
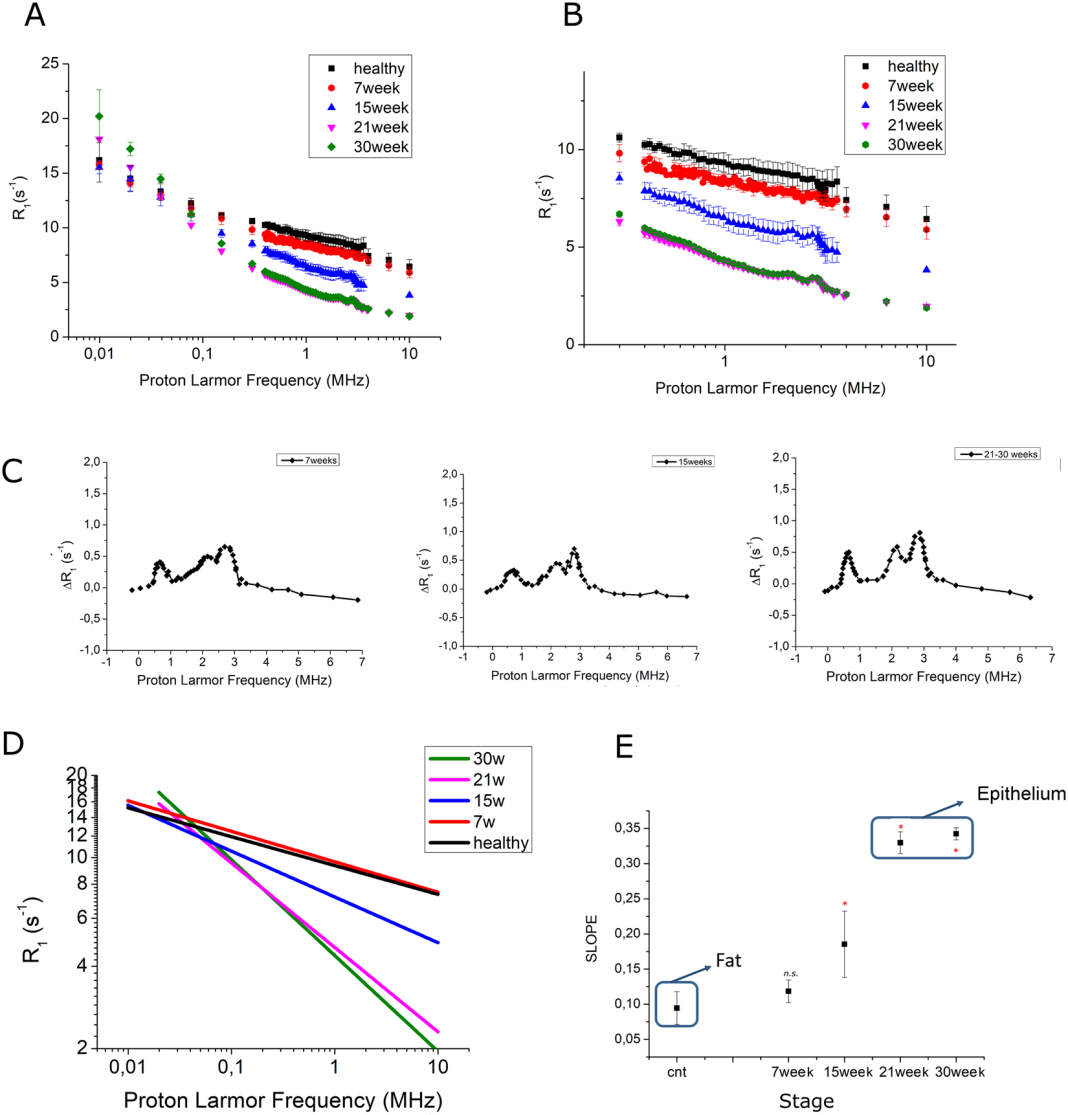
(**A**) NMRD profiles of breast cancer at different stages (7, 15, 21 and 30 weeks) and of control healthy breast tissue (Mean ± SD of at least 8 independent specimens). (**B**) Magnification of the NMRD profiles in the region 0.1–10 MHz range; (**C**) Representative QPs obtained by subtracting background (5–7w, 15w and 21–30w), (**D**) Linear fitting of NMRD profiles (in the log/log scale) and (**E**) slope of the linear fitting for control and NeuT mice at different stage of tumor growth (Mean ± SD) (*P-value < *0.1)*.

The R_1_( = 1/T_1_) data acquired on the FFC spectrometer have been plotted against the applied magnetic field strength which varied, as expressed in terms of Larmor Frequencies, in the 0.01–10 MHz range (Fig. 2A,B).

At low fields (*i.e* B_0_ < 2.4 mT, corresponding to 0.1 MHz) R_1_ increases in the presence of cancer, especially at the advanced stage (Fig. 2A). On the contrary, at higher magnetic fields (*i.e*. > 2.4 mT, corresponding to 0.1 MHz) R_1_ decreases in presence of cancer (Fig. 2B). Overall this finding appears consistent to our current understanding of T_1_ values of tumor tissue at the imaging fields. It is a common understanding that T_1_ of tumor tissue are slightly longer (*ca*.10%) than the corresponding values in healthy ones. However, the herein reported data (Fig. 2) call for a peculiar behavior as the T_1_ difference between healthy and tumor tissue appears much larger.

Likely, the observed behavior is associated to the chemical composition of mammary gland cells that are largely represented by adipocytes, *i.e* by cellular systems in which the protein/fat/water ratio is profoundly altered in respect, for instance, to muscle, epithelium or brain cells. Interestingly, as shown in Fig. 2B (magnification of Fig. 2A), characteristics ^14^N-quadrupolar peaks (^14^N-QPs) can be detected, due to the ^14^N-^1^H cross-relaxation phenomena occurring between water protons and N-H moieties of semi-solid proteins^24–26^. These peaks (at 0.7, 2.1 and 2.8 MHz) are absent in the NMRD profiles of the mammary gland tissue of healthy Balb/c mice but they are invariantly present in the NMRD profiles of NeuT mice at all stages of tumor development, already from the first investigated time point (5–7w). The subtraction of the tissue’s background component allows to detect the occurrence of ^14^N-QPs at all tumor development (starting at the 5–7w stage). Figure 2C reports the QPs region *for representative tissues at 5–7w, 15w and 21–30w*.

Overall, the NMRD profiles display a monoexponential decay when the data are represented in a semilogaritmic graph. Therefore, by translating them in a log/log graph, it has been possible to fit the obtained data with a linear function (Fig. 2D). The slope of straight line connecting R_1_ data for healthy mice is very small (*ca*. 0.1). The slope is maintained small in the presence of tumors at very early stages (5–7 weeks). As the tumors grow, this value increases by reaching the values of 0.12, 0.19, 0.32 and 0.34 at 7w, 15w, 21w and 30w, respectively (Fig. 2E). The small slope value observed for healthy mice appears typical of the presence of a tissue whose main component is still represented by lipids. On the contrary, large slope values (*e.g*. NeuT at 21w and 30w) are typical of the presence of epithelial tissue, as present in breast cancers at later development stages. The transition zone (NeuT at 7w and 15w) are composed by samples with a mixture of fat and epithelial tissues.

^14^N-QPs are not present in healthy breast tissue while they are evident in mice from 5–7w (Fig. 2A,B). Inspection into the NMRD profiles showed that QPs’ amplitude are relatively similar at the different tumor stages. Thus the appearance of the QPs in the NMRD profiles appears as a unique tumor biomarker. It is remarkable that ^14^N-QPs are already present in specimens at a very early stage of tumor development (5–7w).

**Figure 3:**
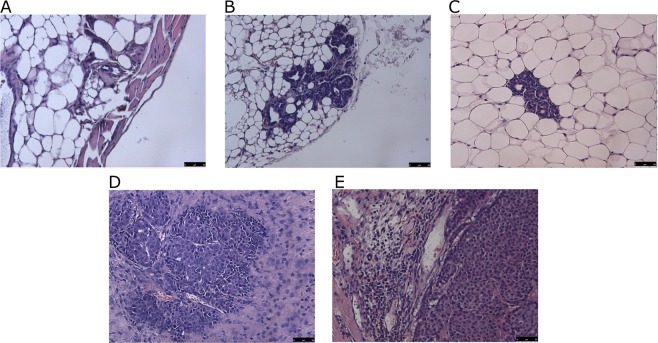
Representative Hematoxylin/Eosin staining of breast tissue at different stages: (**A**) healthy Balb/c mouse, (**B**) NeuT mouse at 7w, (**C**) NeuT mouse at 15w, (**D**) NeuT mouse at 21w and (**E**) NeuT mouse at 30w (Magnification 20x).

NMRD data were correlated to histological analysis. NeuT mammary glands are characterized by a mosaic of different histological phenotypes (simple hyperplasia, atypical hyperplasia, carcinoma *in situ*, invasive cancer), whose relative abundance depends on the tumor stage (Fig. 3)^29,30^.

In 5–7w mice, they are mainly composed by adipose tissue, with some foci of simple hyperplasia (Fig. 3B). In 15w mice, simple hyperplasia plus atypical hyperplasia represents the larger part of the gland (Fig. 3C). At 21w, most of the gland is composed by simple and atypical hyperplasia with the occurrence of large foci of carcinoma *in situ* (CIS) (Fig. 3D). Finally, in 30w mice, more than half of the gland is composed by CIS and invasive carcinoma (Fig. 3E).

**Figure 4:**
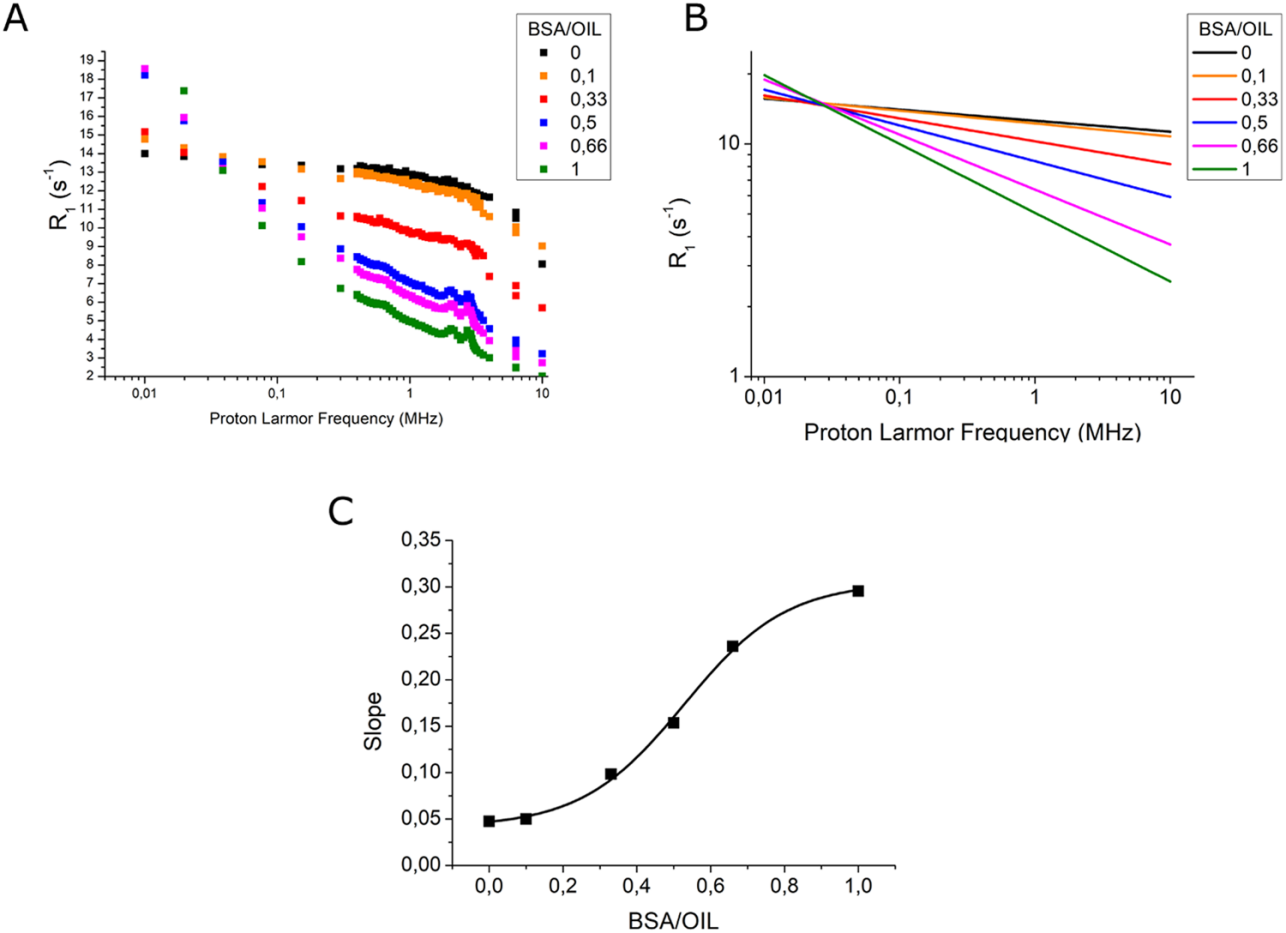
(**A**) NMRD profiles of specimens containing cross-linked BSA and oil at different ratio; (**B**) Linear fitting of NMRD profiles (in the log/log scale); (**C**) Slope of the linear curves *vs*. BSA/oil ratio.

In order to validate results obtained *in vivo*, mammary tissue has been mimicked *in vitro* by preparing specimens containing cross-linked albumin (representative of epithelial tissue, showing in the QPs, Fig. 4A *green points*) and oil (representative of fat tissue, not showing quadrupolar peaks, Fig. 4A *black points*) at different ratios (*i.e*. BSA/oil is 0, 0.1, 0.33, 0.50, 0.66, 1). NMRD profiles have been acquired and analyzed to obtain insights into the slope of R_1_ *vs*. proton Larmor frequency (log/log curve) (Fig. 4B,C). In analogy to what happens for healthy mammary tissue, the specimen containing only oil displays an overall high and constant R_1_ value (Fig. 4A). QPs are not present and the curve slope is low (Fig. 4B,C). Upon increasing the amount of cross-linked BSA, there is an increase of the slope of the curve. This slope is proportional to the molar fraction of BSA in the analyzed specimen, with a behavior which strongly resembles the one reported for *ex vivo* excised tissues (Fig. 4C). QPs are present in all specimens containing BSA, also when only 10% of the specimen is composed by BSA. Therefore, the occurrence of cross-linked albumin is well detectable by the presence of peaks (*on/off response*).

The access to the cross-linked BSA/oil specimens has allowed an in-depth evaluation of the role of the M_z_-(Magnetization) acquisition protocols on the appearance of QPs in the NMRD profiles. It has been found that, upon increasing the τ values and analyzing the M_z_-recovery curve as a bi-exponential function, the intensities of QPs in the *ex vivo* mammary tumor containing tissues did not change much. The acquisition of the M_z-_recovery curve in the latter specimens was carried out considering a mono-exponential behavior for calculation of the T_1_ values as it is routinely done in FFC-relaxometry studies^31^.

## Discussion

In the case of Breast Cancer, it is well established that a positive outcome is markedly dependent on the time it is diagnosed. Therefore, much attention is devoted to methods that allow detection at an early stage, even when no macroscopic sign of disease is present^1,4^.

Herein, a transgenic mouse model of breast cancer, BALB-NeuT, has been investigated^22^. This model recapitulates human breast cancer development, including mammary simple hyperplasia, atypical hyperplasia, CIS and invasive breast cancer. Moreover, BALB-NeuT mice overexpress the activated form of the ErbB2 (Her/2-neu) oncogene, whose amplification is typically observed in 20–30% of human breast tumors^32,33^.

Herein reported i*n vivo* results showed that breast cancer is detectable in T_2w_ (T_2_-weighted) MR images only from 15w, when main portions of the gland are characterized by hyperplasia (simple or atypical).

The mammary glands visualized *in vivo* by MRI were analyzed *ex vivo* by recording the FFC-NMRD profiles. A characteristic feature of the NMRD profile of healthy mammary gland tissue is the high value of R_1_ over all the range of investigated B_0_ fields. This finding appears to be associated to the overwhelming presence of adipocytes with their typical large lipidic content. In these cells, water content is scarce and cytoplasmatic proteins occupy a limited space between the lipidic drops and the cellular wall. The constancy of R_1_ may be accounted in terms of the fact that the proton signal is essentially generated by a dominant lipidic component. Interestingly the hyperplasia step removes this constrain and the newly formed cells display a ^1^H relaxometric response that is driven by the water content and dynamics, as witnessed by the higher slope of the normalized R_1_ function *vs*. B_0_ and the appearance of the ^14^N-QPs.

The results from the *ex vivo* mammary tumor specimens have been validated *in vitro* by acquiring NMRD profiles of samples containing different ratios of cross-linked BSA mixed and oil. Interestingly, an analogue behavior as the one observed for mammary tissue, either in terms of shape and QPs appearance, is occurring in the *in vitro* experiments on the mixtures of BSA and oil.

Therefore, FFC-NMRD profiles appear to be well suitable for reporting about breast tumor onset and development. In normal healthy mammary gland tissue, no ^14^N-QP is present. On the contrary, three peaks are clearly detected (0.7, 2.1 and 2.8 MHz) at each stage of cancer growth, already at the very early stage (5–7w). On this basis, the presence of ^14^N-QP can be considered a proper biomarker of tumor onset, with an *on/off* effect. This behavior is associated to the transition from normal mammary gland tissue, essentially composed by adipose tissue, to epithelial-like tissue in the presence of cancer. In epithelial-like tissue, immobilized proteins are present, that are responsible for the occurrence of the ^1^H-^14^N cross relaxation mechanism at the basis of the generation of quadrupolar peaks in the longitudinal water proton relaxation rates (R_1_).

The observation that no significant quantitative difference in the QPs’ parameters is present at different stages of tumor development appears to be independent on the acquisition protocol of NMRD profiles.

In fact, by using the same time intervals (τ values) in the acquisition of M_z_-recovery curve, it happened that the contribution from different T_1_ value is not well matched with the relative concentration of those proton sources. Support to this explanation has been gained in the *in vitro* experiments on cross-linked BSA/oil specimens by extending the number of τ values and analyzing the M_z_-recovery curve as a bi-exponential function. It has been found that the intensity of the QPs is, as expected, to some extent modulated by differences in the protocol used for the acquisition of the NMRD.

By analyzing the overall shape of the NMRD profiles, more insights into the tissue characteristics can be gained. The linear fitting of log/log NMRD profiles has been shown to be particularly useful for establishing differences among the different tumor stages as each stage appears characterized by a peculiar slope of the linearized NMRD profile. In particular, the slope appears very small for healthy tissue and increases with the progression of the disease.

It follows that, on one hand the occurrence of ^14^N-QPs provides evidence for the presence of tumor cells and, on the other hand, the overall NMRD shape provide information on the tumor development stage.

One limitation of this study is related to the fact that it has been only carried out on *ex vivo* breast specimens, because the currently available FCC-NMRD instruments do not allow the *in vivo* acquisition of NMRD profiles of mammary tumors. Nevertheless, the overall T_1_ of tissues, analyzed at different tumor stages, showed to add relevant information in the characterization of mammary gland tumor, making possible the early detection (5–7w) and further characterizations of tumor phenotypes.

On this basis, it appears evident that FCC-NMRD may have a very high potential in cancer diagnosis and in the establishment of tumor signatures.

Next steps will have to deal with the development of variable magnetic field MRI scanners, able to provide 3D images of tissues at variable and low (<0.24 T) magnetic fields. One may expect that, by using this approach, it will be possible to perform the acquisition of two images (*e.g*. one at B_0_ = 0.24 T and another one tenth of this value) whose ratio will parallel the slope herein detected from NMRD profiles, thus reporting about the presence of the tumor and, eventually, the development stage.

## Conclusions

The herein reported results show that FFC-NMRD profiles provide useful insights for the early detection and characterization of breast cancer. Two complementary information can be gained from the analysis of the profiles. The onset of ^14^N-QPs is highly specific of the presence of tumor, thus providing an unambiguous *on/off* information. In fact, QPs peaks are specifically present only when tumor cells are present in the breast tissue and, more importantly, they show up already at a very early stage of tumor development.

Another relevant information is gained by looking at the overall profile’s shape. The overall slope of the NMRD profile reports about the tumor stage, making it possible to distinguish different tumor phenotypes.

In summary, one may conclude that FFC relaxometry of tumor containing tissue can be a tool for the very early diagnosis and characterization of breast cancer. This observation provides a strong support to the role that FFC-MRI scanners may have in the diagnostic arena and prompts the development of FFC-NMR instruments for *in vivo* acquisition of the T_1_ dependence from the applied magnetic field imaging and diagnosis^18,20^.

The herein reported results show that new avenues to breast cancer diagnosis without the use of exogenous contrast agents are possible eventually using MRI scanners working at magnetic fields lower than the ones currently available in clinic (*i.e*. B_0_ < 0.2 T).
